# Surface ruptures database related to the 26 December 2018, M_W_ 4.9 Mt. Etna earthquake, southern Italy

**DOI:** 10.1038/s41597-020-0383-0

**Published:** 2020-02-07

**Authors:** F. Villani, S. Pucci, R. Azzaro, R. Civico, F. R. Cinti, L. Pizzimenti, G. Tarabusi, S. Branca, C. A. Brunori, M. Caciagli, M. Cantarero, L. Cucci, S. D’Amico, E. De Beni, P. M. De Martini, M. T. Mariucci, A. Messina, P. Montone, R. Nappi, R. Nave, D. Pantosti, T. Ricci, V. Sapia, A. Smedile, R. Vallone, A. Venuti

**Affiliations:** 1 Istituto Nazionale di Geofisica e Vulcanologia, Rome, Italy; 2 Istituto Nazionale di Geofisica e Vulcanologia, Catania, Italy

**Keywords:** Volcanology, Seismology, Tectonics

## Abstract

We provide a database of the surface ruptures produced by the 26 December 2018 Mw 4.9 earthquake that struck the eastern flank of Mt. Etna volcano in Sicily (southern Italy). Despite its relatively small magnitude, this shallow earthquake caused about 8 km of surface faulting, along the trace of the NNW-trending active Fiandaca Fault. Detailed field surveys have been performed in the epicentral area to map the ruptures and to characterize their kinematics. The surface ruptures show a dominant right-oblique sense of displacement with an average slip of about 0.09 m and a maximum value of 0.35 m. We have parsed and organized all observations in a concise database, with 932 homogeneous georeferenced records. The Fiandaca Fault is part of the complex active Timpe faults system affecting the eastern flank of Etna, and its seismic history indicates a prominent surface-faulting potential. Therefore, this database is essential for unravelling the seismotectonics of shallow earthquakes in volcanic areas, and contributes updating empirical scaling regressions that relate magnitude and extent of surface faulting.

## Abstract (summary)

**Table:** 

Measurement(s)	coseismic surface rupture • surface rupture kinematics • surface rupture displacement • surface rupture location
Technology Type(s)	field survey • GPS navigation system
Factor Type(s)	offset • strike • angle • length • latitude • longitude • elevation
Sample Characteristic - Environment	volcanic field
Sample Characteristic - Location	Island of Sicily • Mount Etna

Machine-accessible metadata file describing the reported data: 10.6084/m9.figshare.11673027

## Background & Summary

**Fig. 1:**
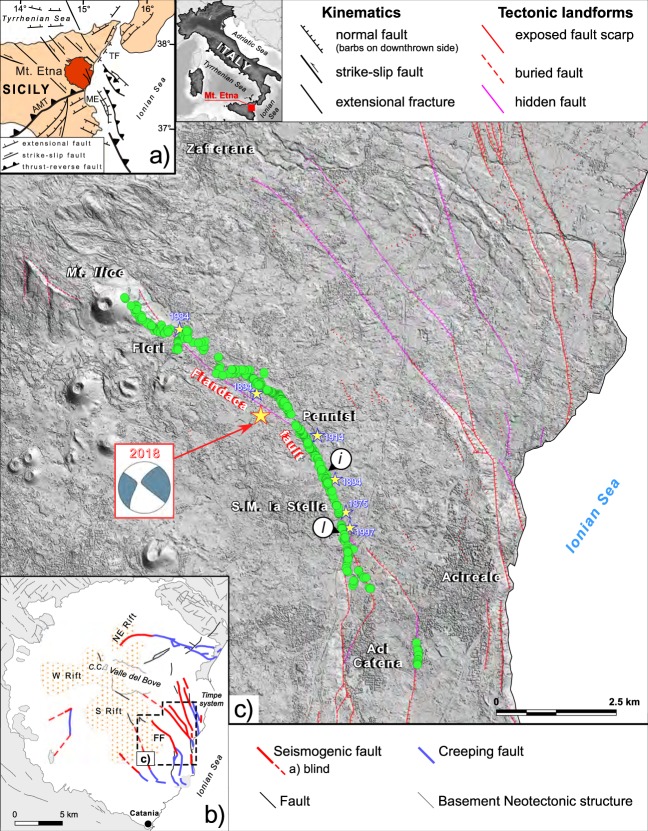
Structural setting of the area struck by the 26 December 2018 Mw 4.9 Mt. Etna earthquake. (**a**) location map showing the Mt. Etna volcano in the framework of the Apennines-Maghrebian thrust-belt; (**b**) sketch of the main active fault-systems on Mt. Etna (FF: Fiandaca Fault); (**c**) detail of the Fiandaca Fault showing the surveyed observation points reported in the present database (green circles), and the focal mechanism of the 26 December earthquake; the main historical earthquakes related to the Fiandaca Fault are reported as pale yellow stars labelled with the year of occurrence. Small labelled circles refer to observation points reported in Fig. 2.

Mt. Etna in eastern Sicily (Fig. 1) is a polygenetic basaltic volcano that started forming about 500 ka, at the outer edge of the Apennines-Maghrebides thrust-belt^1,2^. The present-day volcanic dynamics exhibits a permanent activity from the summit craters and recurrent flank eruptions, paired with a diffuse volcano-tectonic seismicity consisting of frequent small- events (local magnitude M_L_ < 3) with shallow hypocentral depths (h < 5 km)^3,4^. Larger earthquakes up to M_L_ ~5 caused significant damage on the densely urbanised flanks of the volcano, thus they represent a relevant source of hazard^5^. Overall, local geodynamic processes at Mt. Etna are due to the interaction between regional tectonic stresses, volcano dynamics (inflation due to magma uprising, dyke intrusions) and flank instability^6–8^. Most of the seismically active faults are located in the eastern flank of the volcano: the Timpe Faults System consists of a number of up to 9 km-long parallel east-facing step-faults (Fig. 1). These active structures exhibit different fault behaviour along strike, varying from purely stick-slip to stable-sliding movements by creep mechanism, with slip rates ranging from ~1 mm/yr to 2.3 cm/yr^9^. Historical damaging earthquakes characterized by surface ruptures in this area are well documented, demonstrating that faults may rupture their entire length (*i.e*. following a characteristic earthquake style) or smaller individual segments^10^.

The most recent activity of the Fiandaca Fault followed the 24 December 2018 eruption, when a 2 km-long fracture opened in the upper part of the volcano due to a large dyke intrusion^11^. Such process was accompanied by an intense seismic swarm: the local network of INGV recorded about 2,000 earthquakes until the end of the year and >20 events with M_L_ > 3 occurred by the end of February 2019 (details available at http://sismoweb.ct.ingv.it/maps/eq_maps/sicily/catalogue.php). Most of those events clustered around the summit craters; however, a few events are clearly related to the activation of a number of faults along the eastern flank of the volcano. In the final phase of the eruption (ended on December 27), seismicity migrated eastward with a significant seismic release that culminated on 26 December 2018, 02:19 UTC, when a moment magnitude M_W_ 4.9 earthquake nucleated on the Fiandaca Fault, characterized by a main slip patch at ~0.4–1 km depth with peak slip of 0.7 m^12^. Heavy damage due to ground shaking and surface displacement affected buildings, roads and other man-made structures along the fault trace, leaving a thousand of people homeless^13^. Surface faulting extended for ~8 km along strike, rupturing entirely the Fiandaca Fault similarly to the 1894 earthquake, whereas other events (occurred on years 1875, 1907, 1914, 1984 and 1997) produced faulting along different but shorter segments of the same structure^10^. The historical surface displacements present a main right-lateral kinematics with local significant extensional component, and maximum slip of 0.20 m.

Soon after the 26 December earthquake, the EMERGEO Working Group by INGV organised field teams to collect data on the earthquake ruptures before they could be modified by natural or artificial processes^14^. This led to a detailed survey to map the surface effects, measuring orientation and displacement of the ruptures^15^.

Here we describe the data collected by the field campaigns and their processing and organization in a GIS database.

In a purely tectonic domain as the Apennines (Italy), surface faulting is typically associated with earthquakes larger than magnitude M ~6, on faults having centennial to millennial recurrence times^16–19^. Surface faulting on active volcanoes, documented for large (M > 7) earthquakes^20^, is relatively uncommon since the high geothermal gradient and the presence of shallow magma chambers (i.e. melt rocks) may hamper rupture propagation^21,22^. However, in a volcano-tectonic setting such as Etna, surface ruptures can be associated even with moderate-sized earthquakes having recurrence times of a few years^5^. This circumstance allowed us to collect new data sets able to improve empirical scaling relationships for small events^23–26^. Moreover, the collected data are precious to fulfil the primary objective of Fault Displacement Hazards Analysis (FDHA), aimed at quantifying the spatial distribution and magnitude of surface displacements caused by earthquake faulting and their impact on structures and critical facilities. Our database is indeed a contribution to the worldwide compilation of surface-faulting earthquakes^27,28^, especially since moderate magnitude volcano-tectonic events are not sufficiently represented yet.

## Methods

The database described in this paper contains concise geological and structural data describing the surface rupture, and its structure is based on metadata and data records that are defined as variables of different types. The chosen methodological approach consists of the following steps: (1) field survey and data acquisition, (2) data classification and analysis, (3) data screening and formatting, (4) final quality check.

### Field survey and data acquisition

The field survey of the surface geological effects produced by the 26 December 2018 M_W_ 4.9 earthquake has been carried out according to the classical morphotectonic and structural geology methods^29^, routinely adopted by the EMERGEO Working Group^30^. This approach focused on systematic surveys in the epicentral region documenting in detail any geomorphic and structural elements related to surface faulting on both natural and human environment. These include: newly-formed ground ruptures and fault scarplets affecting soils, consolidated/loose volcanic deposits and massive lavas; fractures and dislocations affecting a variety of man-made features (*e.g*. buildings, roads, fences, etc.), requiring attention to elude false interpretation about block-rotation, cumulative displacement etc. In order to avoid the alteration or obliteration of the earthquake-related features due to the winter weather conditions and to the dense urbanization in this region, several field teams were organized to carry out the survey more quickly.

Furthermore, because of the complex behaviour of the Fiandaca Fault (stick-slip and creeping), an immediate mapping of the coseismic ruptures has been crucial to unravel the real amount of slip produced essentially by the 26 December 2018 mainshock. Systematic field measurements have been collected between 28 December 2018 and 18 January 2019. With regard to this difficult task, we point out that the assessment of coseismic slip through a geological survey (that is, within a few minutes from the origin time of the earthquake) is impractical, and any surface measurement contains a portion of afterslip, whatever its relative magnitude, even in a complex combination with background creeping processes^6–10^. However, we can consider those surface ruptures as representative of the coseismic surface slip (*i.e*. caused by the upward seismic rupture propagation occurred during the 26 December M_W_ 4.9 mainshock) for several reasons. First, there is widespread eyewitness of freshly formed surface ruptures immediately following the earthquake. Second, our first quick survey performed in the early morning of 26 December 2018, constrained the overall length of the rupture and the zones of peak slip. Third, the relatively short time-span (<3 weeks) for offset data collection, reduces the contribution of significant post-seismic slip. Fourth, by comparing coseismic slip at shallow depth^12^ and at the surface, we found a good agreement, and the amount of afterslip is likely as small as our measurement uncertainty (±1 cm).

Notwithstanding difficulties to access several sites, the field work guaranteed a dense sampling of the coseismic effects (observation points are on average nearly 10 m-spaced along the whole extent of the surface rupture), thus enabling a reliable reconstruction of the structural pattern and offset distribution.

All structural and offset data have been collected through digital mobile devices equipped with a specific software (Rocklogger^©^ mobile app, www.rockgecko.com) employing accelerometer, gyroscope, electronic magnetometer and global position system (GPS) to determine the exact orientation and position in space of the observed coseismic features. The absolute elevation of measurement points has been subsequently extracted from a 2-m grid Lidar digital terrain model (DTM) available from Ministry of the Environment and for Protection of the Land and Sea website (http://www.pcn.minambiente.it/).

This kind of approach allowed a quick and accurate data collection and, at the same time, a real-time data sharing with the Geology and Geotechnologies Laboratory at INGV in Rome^14^. In the field, the mobile devices have been used to measure the position of the observation point and the orientations of planes (dip angle, dip direction, strike) and lines (slip vector trend, slip vector plunge) whereas measuring tapes have been used for linear measurements (*e.g*. length of surface trace, opening, vertical offset and net offset of the ruptures). Finally, strike measurements of ruptures having appreciable vertical separation have been collected adopting the right hand rule (*i.e*. looking to the strike direction, the plane dips to the right of the observer).

During the survey and data analysis, we took advantage of available detailed tectonic and geologic maps of Mt. Etna region^2,9^. The Fiandaca Fault is mapped as a ∼13 km-long, late Quaternary right-lateral fault-system located in the southernmost part of the Timpe Faults System, on the eastern flank of Mt. Etna (Fig. 1). It is characterised by coexisting seismogenic and creeping behaviour along strike: the locked section shows evidence of coseismic surface faulting events during past earthquakes^10^, and ruptured again with the 26 December 2018 earthquake^14^.

**Fig. 2:**
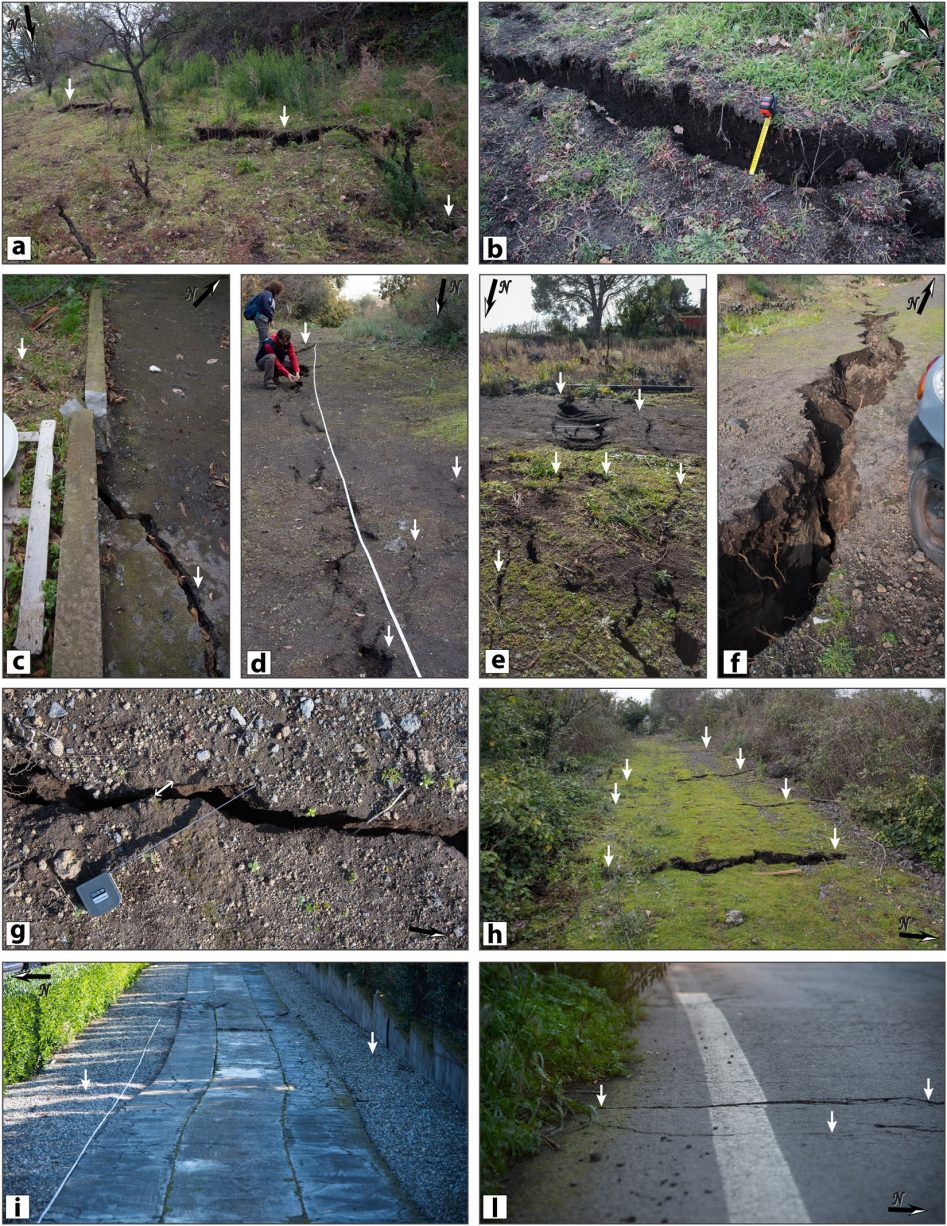
Field examples of the 26 December 2018 earthquake surface ruptures along the Fiandaca Fault. Location of each picture is reported in Fig. 1, and the coordinates (decimal degrees) are given in the following: (**a**) (37.6628N, 15.0907E); (**b**) (37.6627N, 15.0908E) (**c**) (37.6595N, 15.0941E); (**d**) (37.6506N, 15.1153E); (**e**) (37.6490N, 15.1179E); (**f**) (37.6465N, 15.1225E); (**g**) (37.6487N, 15.1183E); (**h**) (37.6417N, 15.1260E); (**i**) (37.6333N, 15.1317E); (**l**) (37.6209N, 15.1366E).

We collected hundreds of georeferenced pictures as well, in order to document in detail the rupture characteristics. Some photographic examples of the main coseismic effects observed in the field are included in Fig. 2. A comprehensive photographic collection of the most relevant surface ruptures has been published by EMERGEO Working Group^31^.

### Data classification and analysis

We have applied a classification of the surveyed ruptures similar to the scheme adopted by the Open EMERGEO Working Group^32,33^ during the recent seismic sequence that hit Central Italy in 2016. The ruptures in most cases are characterized by perceivable opening and an evident oblique kinematics. As reported in detail in *Data Records*, we were able to document a wide spectrum of kinematic features, denoting a local high degree of complexity of the surface faulting.

Ground ruptures occur as discrete fracture systems with lengths ranging from 0.5 m up to 530 m, mostly organized in sub-parallel strands with prevailing *en-échelon* left-stepping arrangement, that define a main deformation zone from a few meters to more than 50 m wide. The main rupture extended almost continuously from the village of Fleri to the southeast, downslope for a length of ~8 km^14^ (Fig. 1). Some off-fault ruptures, probably related to triggered faulting or post-seismic creep, have been documented to the south: they define an underlap zone of about 1.3 km, so that overall the surveyed rupture system is ~10 km-long.

### Screening and formatting

The raw data have been collected in the field as comma-separated values (CSV) through the use of the Rocklogger© mobile app. The third step of the work consisted in the screening of the collected data to proceed with their formatting^30,33^ by selecting a number of fields that synoptically describe our data. Firstly, we performed a check of the database to delete duplicate records and other typing errors accidentally done during fieldwork.

Then we assigned a progressive ID to each record sorting the dataset according to the acquisition data/time, and then converted the files into an ESRI shapefile (SHP) on the ArcGIS© suites, so they can be plotted, explored and edited by a standard query language (SQL). After a final quality check, the SHP file has been converted into the text (TAB) file provided in this work.

## Data Records

**Fig. 3:**
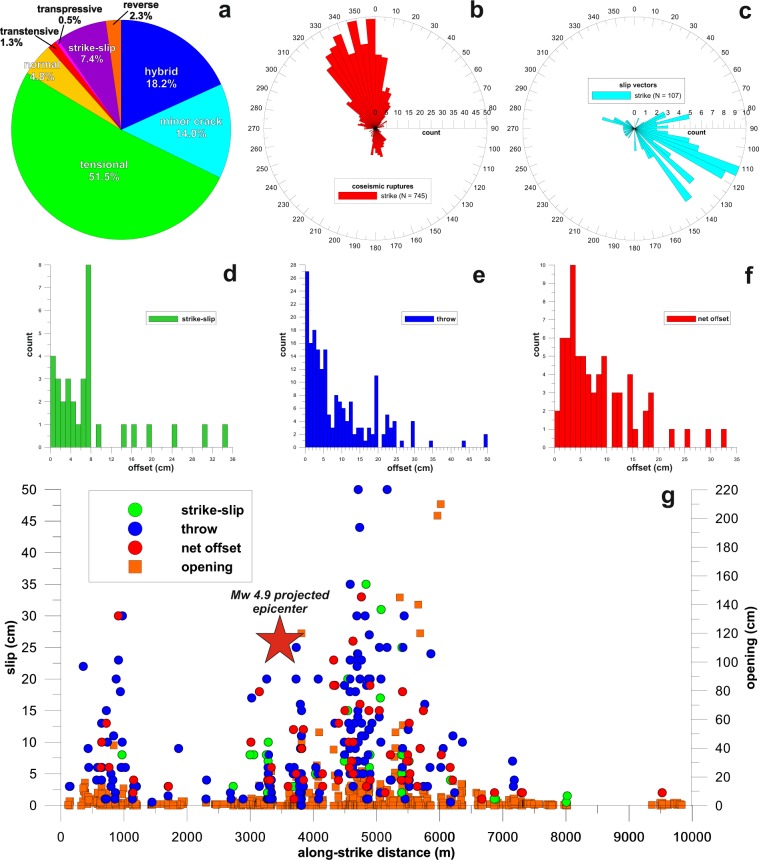
Statistical properties of the surface ruptures of the 26 December 2018 earthquake along the Fiandaca Fault. (**a**) pie diagram with the kinematic classification of the ruptures and their frequency of occurrence; (**b**) rose diagram of coseismic ruptures strike (bin size = 5°); (**c**) rose diagram of slip vectors strike (bin size = 5°); (**d**) frequency histogram of strike-slip; (**e**) frequency histogram of throw; (**f**) frequency histogram of net offset; (**g**) measured coseismic surface offset projected onto a common baseline paralleling the Fiandaca Fault (the origin of the line is located at the north-western tip of the fault): the red star indicates the projection of the 26 December M_W_ 4.9 earthquake epicentre over the baseline.

The complete dataset here presented is stored in the *Pangaea* repository^34^ as a TXT file (Villani-etal_2019.tab). After final screening and editing, the data output contains 932 records organized into 19 fields. Each record describes a measure at a single (observation) point, with a scheme similar to that used for the database of the coseismic effects following the 30 October 2016 M_W_ 6.5 earthquake in central Italy^33^. The fields have a name and a short name, described as follows:

1. **ORDINAL NUMBER** (short name: Ord No): integer type variable defining the object identifier;
2. **DATE/TIME** (short name: Date/Time): date type variable indicating the date of data collection, in the format yyyy-mm-ddThh:mm (year, month, day, hour, minute);
3. **LATITUDE** (short name: Latitude): double type variable indicating the latitude of the observation, in decimal degrees (dd.mmmmmm, six decimal places) within a WGS_1984 Geographic Coordinate System;
4. **LONGITUDE** (short name: Longitude): double type variable indicating the longitude of the observation point, in decimal degrees (dd.mmmmmm, six decimal places) within a WGS_1984 Geographic Coordinate System;
5. **ELEVATION** (short name: Elevation): double type variable indicating the absolute elevation of the observation point in meters above the sea level (one decimal place); the elevation is extracted from a 2-m grid DEM described in the Methods Section;
6. **Observation** (short name: Obs): text variable indicating the basic category of the geological surface effect observed; for simplicity, only three main categories are defined as follows: 1) “coseismic rupture” (rupture displaying a perceivable offset of the ground surface, on the order of at least few cm); 2) “coseismic slip vector” (lineation defining the direction of coseismic net displacement occurred along a rupture); 3) “hinge” (lineation defining the trend and plunge of the hinge of an anticline or a syncline due to coseismic warping of the ground surface);
7. **Substratum** (short name: Substratum): text type variable including the synthetic description of the lithological nature of the substratum (natural or artificial) where the coseismic effect has been observed;
8. **Angle** (short name: Angle): double type variable indicating the angle of dip of a rupture, measured in degrees (no decimal places);
9. **Direction** (short name: Direction): double type variable indicating the direction of dip of a rupture or sliding surface with respect to the North, measured in degrees (no decimal places);
10. **Strike** (short name: Strike): double type variable indicating the azimuth angle of a rupture direction or sliding surface with respect to the North (British right hand rule), measured in degrees (no decimal places);
11. **Length** (short name: l): double type variable indicating the length of a rupture measured in meters (one decimal place);
12. **Opening** (short name: Opening): double type variable indicating the aperture of a rupture along a horizontal direction perpendicular to the surface trace of the rupture and measured in centimetres (no decimal places);
13. **Throw** (short name: Throw): double type variable indicating the vertical separation of a coseismic rupture measured in centimetres (one decimal place);
14. **Strike-slip** (short name: Strike-slip): double type variable indicating the horizontal separation of a coseismic rupture along the rupture strike direction measured in centimetres (one decimal place);
15. **Offset** (short name: Offset): double type variable indicating the net displacement of a coseismic rupture measured in centimetres (one decimal place) along the slip vector, using piercing points (*i.e*. recognizable cut-offs on both sides of the rupture);
16. **Kinematics** (short name: Kinematics): text type variable describing the relative movement of the two blocks separated by a coseismic rupture (Fig. 3a; Table 1).

**Table 1:** kinematic typologies reported in our database, and offset criteria used for classification of surface ruptures.

kinematic type	opening (cm)	throw (cm)	strike-slip (cm)
hybrid	>1	>1	n.a. or <1
minor crack	≤1	≤1	n.a. or ≤1
normal	<1	>1	n.a. or <1
reverse	<1	>1	n.a. or <1
strike-slip	<1	<1	>1
tensional	>1	<1	n.a. or <1
transpressive	<1	>1	>1
transtensive	≥1	>1	>1
17. **Trend** (short name: Trend): double type variable indicating the direction of the slip lineation in degrees, measured clockwise with respect to the north (range 0°–360°, no decimal places);
18. **Plunge** (short name: Plunge): double type variable indicating the plunge of the slip lineation in degrees, measured with respect to the horizontal (range 0°–90°, no decimal places);
19. **Width** (short name: W): double type variable indicating the width of a complex fracture network at the outcrop scale, measured in metres (one decimal place) in a direction orthogonal to the strike of the bounding ruptures.

Table 2 reports as an example four records from the database.

**Table 2:** Example of four records of the database available on Pangaea repository.

1	2	3	4	5	6	7	8	9	10	11	12	13	14	15	16	17	18	19
Ord No	Date/Time	Latitude	Longitude	Elevation [m a.s.l.]	Obs	Sub-stratum	Angle [deg]	Direction [deg]	Strike [deg]	l [m]	Opening [cm]	Throw [cm]	Strike-slip [deg]	Offset [cm]	Kinematics	Trend [deg]	Plunge [deg]	w [m]
53	2018-12-29T10:15	37.633483	15.131309	329.5	coseismic rupture	landfill	54	87	357	1200	20	4			hybrid			1
54	2018-12-29T10:16	37.633395	15.13132	329.5	coseismic rupture	landfill	68	78	348	1500	8	30			hybrid			1
55	2018-12-29T10:24	37.633324	15.131528	327.8	coseismic rupture	landfill	65	242	152	520	3	1			hybrid			1
56	2018-12-29T10:24	37.633329	15.131551	327.6	coseismic rupture	landfill	70	62	332	520	2				tensional			1

### Statistical data analysis

Figure 3a shows the frequency distribution of the various kinematic typologies of the coseismic ruptures recognized in the field. This information mostly applies to local details and complexities of the rupture, which in some cases differ from the large-scale pattern of the surface faulting. Nearly 50% of the surveyed ruptures are tensional open cracks, whereas about one third of the remaining ruptures display a hybrid kinematics (a mix of opening and oblique-slip) or conversely are characterized by offset below the error of the measurements made through standard tapes (minor cracks). The remaining ruptures display normal, reverse, strike-slip, transtensional or transpressive kinematics. It is worthy to note that the total number of strike-slip kinematic data appears underestimated (Fig. 2c,i,l), due to the difficulty in finding reliable piercing points and/or clear displaced markers.

The rose diagram in Fig. 3b shows the azimuthal dispersion of the coseismic ruptures: 745 measurements indicate two prevailing peaks of strike at N340°–345° and N355°–360°, respectively, but a subordinate trend at N335°–340° is also evident. The overall trend of the small-scale ruptures mimics the mapped trace of the Fiandaca Fault: in particular, the N355° peak fits the fault section between Pennisi and Santa Maria la Stella, while the N345° peak corresponds to the section between Pennisi and Fleri (Fig. 1). However, a local deviation is evident since the overall rupture envelope at the macro-scale consists of tens of smaller strands typically featuring a left-stepping arrangement (*e.g*. Fig. 2d).

The azimuthal distribution of the coseismic slip vectors is shown in Fig. 3c. Most of them indicate a transtensive, right-oblique kinematics with the northeast side down; a few data indicate right-oblique slip with the south-west side down. The slip vectors, analyzed using software for kinematic structural data^35,36^, indicate an average N125° trending slip direction with a gentle plunge (29°) consistent with the general transtensive kinematics of the Fiandaca Fault, consistent also with the focal mechanism of the M_W_ 4.9 mainshock (dextral, oblique slip on a sub-vertical nodal plane trending N308°, see http://cnt.rm.ingv.it/event/21285011/). The rake angle of fault slip was often not measurable or ambiguous, due to the nature of loose deposits and the lack of hard planar fault surfaces; therefore, it is not reported in the database.

The histograms in Fig. 3d,e,f show the frequency distribution of the horizontal offset (i.e. Strike-slip), vertical offset (i.e. Throw) and net offset (i.e Offset), respectively. Although precise measurements of the surface offset were difficult to be made, those plots clearly indicate non-normal distributions, according to the results described for the Norcia earthquake^18^.

The strike slip offset has an average value of 0.09 m with a peak of 0.35 m, while in most cases the throw is lower than 0.01 m, with an average value of 0.10 m and a local maximum value of 0.50 m. The net slip has an average value of 0.09 m, with a maximum of 0.33 m.

In order to depict the general spatial pattern of the surface offset, Fig. 3g represents the projection of the offset data onto a 10 km-long baseline parallel to the trace of the Fiandaca Fault. No smoothing within moving windows is applied to the data and no summing up of offset values for overstepping strands is done: therefore, the plot provides only a rough first-order picture of the along-strike coseismic slip distribution. However, it is evident that the highest offsets are found at 5 km of distance from the northern fault tip, very close (about 1 km) to the mainshock epicentre, and decrease rapidly towards the fault tips, particularly to the south. A significant amount of surface slip also occurred close to the northern tip of Fiandaca Fault, where gravitational effects emphasized the fault displacement^15^.

## Technical Validation

The technical validation of the collected data is not an easy task, since no experiment can be planned to formally validate a coseismic surface effects database. In fact, the output data records are not exactly reproducible, since they are representative of a unique coseismic event. Moreover, they are strictly tied to the period of survey (between 28 December 2018 and 18 January 2019). Any measurement of surface offset performed weeks to months later can lead to significantly different results. This is due to the action of the natural surface processes, which after an earthquake modify the coseismic topographic anomalies by erosion or sedimentation. Other causes of alteration of the surface ruptures are the necessary repair of the affected infrastructures, buildings, agriculture fields. Moreover, post-seismic slip, fault creep and the general dynamics of the volcano-tectonic environment definitively conceal the coseismic surface offsets (see Background & Summary).

Therefore, in the validation procedure we followed the same approach described for the 2016 Norcia earthquake in central Italy^33^. The following considerations provide a qualitative indication about the reliability and consistency of the collected data: (1) the observed effects are similar with the previous coseismic surface faulting events in the study area, well known along the Fiandaca Fault; (2) the spatial distribution of the ruptures, the prevailing right-oblique kinematics and the amount of surface slip are inherently consistent with the seismological and geodetic framework of the 26 December 2018 earthquake (see Background & Summary). In conclusion, we believe that our data may really help to better constrain the slip model of the mainshock fault.

With regard to the validation of the data, we took into account the factors contributing to the reliability of parameters, such as environmental conditions, representativeness of the surveyed ruptures and measurement characteristics. The uncertainty of the measurements mostly depends on the instrument precision and accuracy, calibration, equipment maintenance and, last but not least, field operations.

As for the measurements of planes and lines orientation, in order to reduce the systematic errors, we repeatedly calibrated the digital mobile devices (Samsung Galaxy Note 4) before each survey, following the standard procedure suggested by the Rocklogger© mobile app.

Mobile devices enable quickly collection of a large amount of measurements, providing statistically significant readings of the geologic object that may be compared with measures taken with an analogue compass. Based on previous experience^33^, we found that the average dispersion of the measured values was ±5°. Depending on the standard tapes used, the measurements of fracture opening, horizontal offset, and vertical offset of the ruptures have a precision of ±1 cm. Therefore, ground ruptures with small offset (a few centimetres) are proportionately affected by intrinsic errors in the order of about 10–20%.

GPS-supported mobile devices have position accuracy with a median horizontal error of position fixes ranging from 5.0 m to 8.5 m^37^, which are comparable to that observed for our measurements points.

Finally, as a basemap to cross-check the observation points in ESRI ArcGIS, we used high-resolution images obtained from ESRI World Imagery (http://services.arcgisonline.com/ArcGIS/rest/services/World_Imagery/MapServer) together with Lidar-derived shaded relief images.
